# The genotype-phenotype relationship in multicellular pattern-generating models - the neglected role of pattern descriptors

**DOI:** 10.1186/1752-0509-3-87

**Published:** 2009-09-04

**Authors:** Harald Martens, Siren R Veflingstad, Erik Plahte, Magni Martens, Dominique Bertrand, Stig W Omholt

**Affiliations:** 1 Centre for Integrative Genetics (CIGENE), Norwegian University of Life Sciences, N-1432 Ås, Norway; 2 Department of Mathematical Sciences and Technology, Norwegian University of Life Sciences, N-1432 Ås, Norway; 3 Department of Animal and Aquacultural Sciences, Norwegian University of Life Sciences, N-1432 Ås, Norway; 4 Nofima Mat AS, Osloveien 1, N - 1430 Ås, Norway; 5 Faculty of Life Sciences, University of Copenhagen, 1870 Frederiksberg C, Denmark; 6 Unité de Sensométrie et de Chimiométrie, ENITIAA-INRA, 44322 Nantes cedex 3-France; 7 Systems Biology Centre, University of Warwick, Coventry, CV4 7AL, UK

## Abstract

**Background:**

A deep understanding of what causes the phenotypic variation arising from biological patterning processes, cannot be claimed before we are able to recreate this variation by mathematical models capable of generating genotype-phenotype maps in a causally cohesive way. However, the concept of pattern in a multicellular context implies that what matters is not the state of every single cell, but certain emergent qualities of the total cell aggregate. Thus, in order to set up a genotype-phenotype map in such a spatiotemporal pattern setting one is actually forced to establish new pattern descriptors and derive their relations to parameters of the original model. A pattern descriptor is a variable that describes and quantifies a certain qualitative feature of the pattern, for example the degree to which certain macroscopic structures are present. There is today no general procedure for how to relate a set of patterns and their characteristic features to the functional relationships, parameter values and initial values of an original pattern-generating model. Here we present a new, generic approach for explorative analysis of complex patterning models which focuses on the essential pattern features and their relations to the model parameters. The approach is illustrated on an existing model for Delta-Notch lateral inhibition over a two-dimensional lattice.

**Results:**

By combining computer simulations according to a succession of statistical experimental designs, computer graphics, automatic image analysis, human sensory descriptive analysis and multivariate data modelling, we derive a pattern descriptor model of those macroscopic, emergent aspects of the patterns that we consider of interest. The pattern descriptor model relates the values of the new, dedicated pattern descriptors to the parameter values of the original model, for example by predicting the parameter values leading to particular patterns, and provides insights that would have been hard to obtain by traditional methods.

**Conclusion:**

The results suggest that our approach may qualify as a general procedure for how to discover and relate relevant features and characteristics of emergent patterns to the functional relationships, parameter values and initial values of an underlying pattern-generating mathematical model.

## Background

### Modelling phenotypic variation in biological pattern generation

The whole development process of higher organisms can be mathematically conceptualised as a recursive mapping - i.e. successive cell differentiations leading to a sequence of unfolding patterns at many different spatiotemporal scales, each pattern defining the context for further differentiation and thus for subsequent patterning processes. A deep understanding of what causes the phenotypic variation arising from such patterning processes cannot be claimed before we are able to recreate this variation theoretically by what we call *causally cohesive genotype-phenotype models *(cGP models) [1]. Unlike the broader class of mechanistic mathematical models describing how complex biological phenotypes arise from the interactions of lower-level systemic entities, cGP models are distinguished by linking together (cohering) the individual's genotype and its phenotype in a causal mathematical structure. cGP models thus allow the construction of genotype-phenotype maps, i.e. mappings predicting the phenotype associated with a given genotype based on what we know about the regulatory anatomy of a given biological system.

Irrespective of the level of biological resolution of a cGP model, genetic variation has to be represented as parametric variation. In a genotype-phenotype-map perspective one is thus interested in getting a clear understanding of the mappings between genotype parameter space and the generated phenotypic space. However, in the context of multicellular patterning models it is not trivial to establish this relation. The very concept of pattern implies that what matters is not the state of every single cell, but certain emergent qualities of the total cell aggregate which express relations between the states of subsets of the cells. Thus, in order to set up a genotype-phenotype map in such a spatiotemporal pattern setting one cannot just establish a mapping between domains in parameter space and certain properties of locally defined intracellular and extracellular state variables. *One is actually forced to construct new descriptors of the emergent pattern features, express them in an abstract pattern descriptor space, and establish their relations to properties of the original model*. In addition to leading to a genotype-phenotype map, this approach opens possibilities for additional validation of the model through prediction of higher level and empirically observable properties that are by no means part of the model's premise set, and which represent emergent features of the pattern that are interesting and relevant from the point of view of purpose and objectives of the original pattern-generating model.

**Figure 1:**
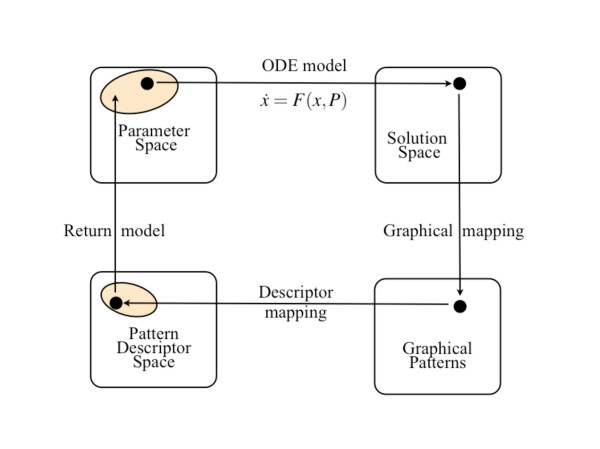
**Traditional and new process of analysis based on simulation studies of a model for pattern formation**. Starting with a point *P *in parameter space (represented by the black dot) given by a chosen set of parameter values, the stable solution *X *is obtained by integrating the dynamical model *dx*/*dt *= *F*(*x*, *P*), *x*(*0*) = *x*^0^. The second step is to represent each *X *by a point in Solution Space (phase space). Using a convenient graphical mapping, *X *defines a Graphical Pattern that can be inspected and analysed visually. So far this is conventional procedure. To analyse and classify the patterns, new concepts, variables and names are necessary to describe the interesting macroscopic and emergent properties of the patterns in a Pattern Descriptor (PD) space. Each pattern descriptor is then given a numeric value according to chosen criteria. This permits the mapping of each point *P *in parameter space onto a point in the PD space in terms of a PD model derived by various multivariate methods (see text). Being approximate and probabilistic in nature, this mapping by the statistical prediction model only indicates a certain domain in parameter space (coloured) which will produce patterns resembling a given pattern.

*There is today no general procedure for how to relate a set of patterns and their characteristic features to the functional relationships, parameter values and initial values of the original pattern-generating model*. Here we propose a multivariate data modelling approach which is based on three major elements: (i) cost-effective computer simulations to probe the high-dimensional parameter space, (ii) informative ways to describe the model's graphical patterns quantitatively as points in a pattern descriptor (PD) space, and (iii) ways to establish two-way mappings between the PD space and the parameter space in terms of a statistically reliable and interpretable statistical prediction model (Figure 1).

We illustrate our approach on a simple mathematical model of pattern generation, and show that even in this case, traditional and intuitive methods prove inadequate to describe and understand its full potential of pattern formation, while our approach provides an overview and reveals unexpected features and their relationship to the model parameters.

### The illustration model

A large number of cell differentiation processes involve the membrane-bound protein Notch which interacts with several transmembrane ligands in neighbouring cells [2-4]. Thus, understanding the diverse functions of Notch is of paramount importance in itself. One patterning mechanism in which Notch is involved is lateral inhibition: a cell-cell interaction whereby a cell developing a specific fate inhibits its neighbours from developing in the same way. Delta is one of the ligands binding to Notch; Delta in one cell binds to Notch in the cells in physical contact, leading to juxtacrine signalling [5]. The multicellular gene network regulating this process is called the neurogenic network [6]. However, as early as 1996 Collier *et al. *[7] presented what can be considered as a simplified multicellular cGP model for Delta-Notch activity in a one- or two-dimensional lattice of discrete cells. In this model the rate of change of Notch activity increases with the average activity level of its ligand Delta in neighbouring cells, while the rate of change of Delta activity decreases with increasing activity level of Notch in the same cell, both relationships being expressed in terms of sigmoidal response functions.

On a two-dimensional lattice of *n *identical hexagonal cells we let *D*_*k *_and *N*_*k*_, *k *= 1,..., *n*, represent the activity level of the two proteins Delta and Notch in cell number *k*. In suitably scaled variables, assuming first-order degradation, and with the standard Hill function representing sigmoid stimulus-responses, the dimensionless model of Collier *et al. *is

(1a)

(1b)

in which *μ *is the ratio between the degradation rates for Delta and Notch,  is the average value of Delta in the six neighbours of cell *k*, and *S *is the standard Hill function *S*(*x*, *θ*, *p*) = *x*^*p*^/(*x*^*p*^+ *θ*^*p*^). Thus, *θ*_D _and *θ*_N _are the thresholds for the action of Delta and Notch, respectively, and *p*_D _and *p*_N _are the corresponding steepness parameters (Additonal file 1: Table S-T1).

The positive feedback loops between any pair of neighbouring cells which these interactions generate, lead to differentiation: high Delta and low Notch activity in a cell favours low Delta and high Notch activity in neighbouring cells, and *vice versa*. On a one-dimensional string of discrete cells, the final, differentiated states are strongly dominated by an alternating pattern: apart from scattered exceptions in which two neighbouring cells both express either Delta or Notch, every second cell expresses Delta and suppresses Notch, and *vice versa*. On a two-dimensional lattice of square cells this behaviour is consistent with the well-known checkerboard pattern. Using standard linearisation methods, Collier *et al. *[7] showed that the system of regular, hexagonal cells possesses three basic 3-periodic patterns consisting of up to three cell types. In our simulations the basic regular patterns appear either by cells expressing Notch surrounded by cells in which Notch expression is inhibited, or *vice versa*.

**Figure 2:**
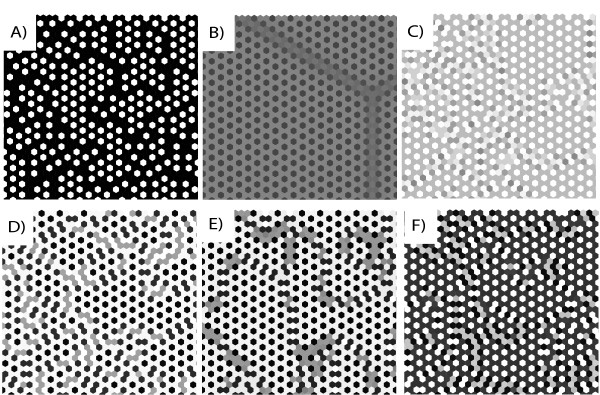
**Selected trimmed equilibrium patterns of Notch activity from the four main pattern classes for the Delta-Notch model on a 50 × 50 hexagonal lattice with periodic boundary conditions**. White means *N *= 0 (no activity), black means *N *= 1 (full activity). All solutions were initiated with different random initial values. The values of *μ*, *M*, and *s *had only minor effects on the final patterns. Other parameter values: **(**A) Class I: *θ*_D _= 0.1, *θ*_N _= 0.1, *p*_D _= 10, *p*_N _= 3; (B) Class II: *θ*_D _= 0.7, *θ*_N _= 0.7, *p*_D _= 3, *p*_N _= 10; (C) Class IV: *θ*_D _= 0.7, *θ*_N _= 0.1, *p*_D _= 3, *p*_N _= 10; (D) Class III: *θ*_D _= 0.7, *θ*_N _= 0.1, *p*_D _= 10, *p*_N _= 3; (E) Class IV: *θ*_D _= 0.7, *θ*_N _= 0.7, *p*_D _= 10, *p*_N _= 10; (F) Class IV: *θ*_D _= 0.4, *θ*_N _= 0.4, *p*_D _= 6, *p*_N _= 6.

However, two-dimensional lattices of real cells are usually rather irregular and do not have periodic patterns. Podgorski *et al. *[8] have combined the model of Collier *et al. *with models for apoptosis and differential adhesion to study the patterning process on irregular cell lattices. But even on a regular lattice of hexagonal cells irregular patterns arise because the system is *frustrated *[9]: it is impossible to satisfy the requirement of opposite expression of Delta and Notch between all pairs of neighbouring cells. If cell 1 expresses Notch, its neighbouring cells 2 and 3 should both express Delta, which causes frustration because cells 2 and 3 also may be neighbours. This results in a multitude of different patterns, sometimes with a high number of different Notch and Delta levels, depending on parameter values and initial conditions (Figure 2).

In fact, our simulations showed that the majority of patterns and pattern features are highly irregular. Initial trial and error simulations for different combinations of parameter values revealed a wide range of different kinds of patterns, and for certain parameter value combinations we observed a large number of different equilibrium levels. Trying to describe these irregularities as combinations of basic regular patterns did not seem to make much sense. Rather, our approach was to try to introduce new concepts and pattern descriptors to classify and describe the irregular pattern as patterns in their own right. It was also obvious that without a systematic, planned simulation design as outlined above, there could be no hope of achieving a sound description and classification of the pattern and reveal their relation to variations in parameter values.

## Results

### General procedure

We combined various explorative principles in order to maximise insight from the computer simulations. Factorial statistical design plans ensured that the parameter space was spanned systematically. Each solution image was characterised by a range of dedicated, quantitative descriptors, and the results interpreted by multivariate data modelling.

We analysed the high-dimensional, non-linear Delta-Notch model on a 2D lattice of hexagonal cells by this approach. For each set of parameter values the system of differential equations was integrated in time until a stable state was reached. The spatial distribution of the stable-state Notch concentrations were plotted as black and white 2D images of the cell lattice for easy visual inspection.

**Figure 3:**
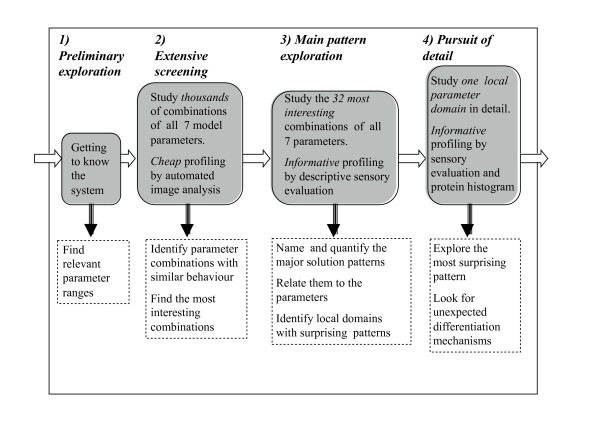
**Method overview: four stages in the investigation of the high-dimensional non-linear dynamic model: (1) Preliminary exploration**. Design: trial-and-error. Analysis: visual inspection of model steady-state solutions, rendered as black and white images of Notch concentrations. (2) Large-scale systematic screening using extensive probing of the parameter space, monitored by computerised profiling by full factorial designs. Relating more than 50 computed image analysis descriptors to 7 known model parameters by regression in latent variables (jack-knifed PLSR). (3) Disciplined investigation using informative but more laborious human sensory descriptive profiling by a reduced factorial design, and relating 15 sensory image descriptors to all 7 model parameters by jack-knifed PLSR. (4) Pursuit of an unexpected discovery in a particular region of parameter space. Dense sampling of *θ*_D _for chosen combination of other parameters, and relating estimated protein distributions and sensory image descriptors to *θ*_D _by graphics.

The exploratory process proceeded through four distinct stages (Figure 3). Together, these four stages were intended to yield maximum new insight into the behavioural repertoire of the model with minimum effort and minimum number of additional assumptions.

(1) The interesting ranges for the model parameters were determined in a preliminary exploration. In this trial-and-error based phase the parameters were changed one at a time, and the solution images inspected informally, to determine values to be used as "high" and "low" for each parameter in the subsequent designs.

(2) Secondly we defined a 2^7 ^full-factorial design and expansions of this in order to explore the parameter space domain found interesting in phase (1). The equilibrium solutions images were submitted to computerised image analysis by standard mathematical filters of various kinds, like gray-tone density and spatial frequency histograms. This profile of many, but *per se *meaningless image descriptors could be related to the seven known model parameter values by well-established multivariate methods from chemometrics, primarily conventional reduced-rank Partial Least Squares Regression (PLSR) [10] with optimal rank determined by cross-validation as described in [11,12]. This permitted us to identify parameter combinations which could be ignored in the main explorative experiment because they had little or no effect on the solution patterns.

(3) In the main experiment we employed a more informative, but also more laborious solution profiling. It consisted of a fractional factorial design created in order to span the range of all parameters and their combinations that were found to have a clear effect in (2), by means of a minimum number of numerical simulation solutions. Due to the insight obtained from our previous screening, our new design could be reduced to only 32 selected combinations of a high or low value of each of the seven model parameters, with six non-informative (no patterning) solutions replaced by two replicates of an intermediate parameter combination with independent random starting value sets, each assessed independently in three sensory parallels (see Additional file 1).

Human sensory descriptive analysis was used for image description. It provides systematic, inter-subjective description in intelligible terms with high repeatability. Such sensory descriptive analysis is standard procedure in food science [13] and also used for describing and comparing e.g. perfumes or the quality of MRI images [14]. Here we used it for assessing printed images of outcomes from the model of cell differentiation. A panel of professional human assessors first developed adequate terminology, and was then trained in describing and comparing a set of images of steady-state solutions from the simulations with respect to these descriptor terms [13,15]. During this process each assessor's subjective "private language" was replaced by a common "inter-subjective" set of terms which was agreed upon by all persons involved, both with respect to what it means qualitatively and to how it should be reported quantitatively. The sensory analyses are detailed elsewhere [16].

The resulting tables of profile data were modelled by multivariate "soft modelling" based on reduced-rank Partial Least Squares (PLS) regression [10-12]. The main covariation patterns among the observed descriptors were identified and related to the chosen model parameter values in a subspace-model defined by statistically significant covariance eigenvectors. Cross-validation was used to distinguish between phenomena with predictive validity and apparent noise effects. This made it possible to find and interpret the main patterns of co-variation while ignoring minor details that otherwise would have lead to over-parametrisation.

The 32 image profiles could thereby be mapped onto points in the PD space, and PLSR was used for further analysis of the descriptors and their relation to the model parameters. Figure 2 shows examples of pattern classes thus identified.

(4) Finally, we pursued unexpected pattern types discovered in the main experiment (3) in more detail in a final follow-up experiment, involving a dense sampling of the model parameter *θ*_D _for a few fixed combinations of the other parameters. The solutions were characterised by both sensory descriptive analysis and by a computational method by which the frequency of the observed Notch concentration levels were recorded at each parameter value. (See Additonal file 1 for documentation of the research process according to Figure 3.) In the following we summarise the main results.

### Pattern Descriptor Space and Pattern Descriptor Model

**Figure 4:**
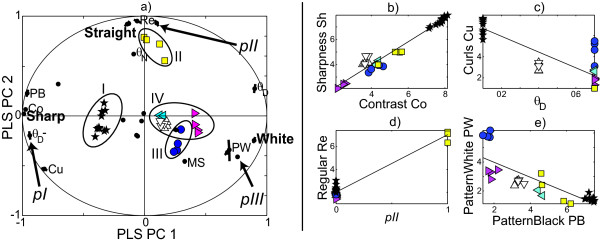
**(a) Main co-variations from sensory exploration (3) mapped by cross-validated PLSR bi-plot in which the design parameters *θ*_D_, *θ*_N_, *p*_D_, *p*_N_, *μ*, *M*, and *s *and selected interactions are related to the sensory image descriptors for 32 solution images from the fractional factorial design, plotted in the subspace of Pattern Descriptor Space spanned by the two first PLS components *PC*_1 _and *PC*_2 _(orthogonal linear combinations of the 12 sensory descriptors)**. Sensory descriptor variables, design parameters and 32 solution score indicators are positioned according to their correlations to *PC*_1 _and *PC*_2_. The origin represents zero correlation; outer ellipsis represents locus of variables with variance explained 100% by *PC*_1 _and *PC*_2_. Variables close to the ellipsis and close to (or opposite) each other are positively (or negatively) correlated to each other. Only the most salient variables are marked. Some correlations are illustrated in (b)-(e). Ellipses show approximate boundaries of the four classes I-IV; the coloured markers represent the classes. (b) Values of *Sharpness vs. Contrast *for the 32 images, *r *= 0.98. (c) *Curls vs. *θ_D_, *r *= -0.84. (d) *Regular vs. *the four-factor interaction term *pII *= [*θ*_D_+, *θ*_N_+, *p*_D _≠ *p*_N_], i.e. *θ*_D _is high,*θ*_N _is high, and *p*_D _is high for low *p*_N _and *vice versa*, r = 0.98. (e) *PatternWhite *and *PatternBlack *are anti-correlated, *r *= -0.82. Plot symbols: black asterisk = class I, yellow square = class II, blue circle = class III, cyan/magenta/white triangles = class IV (see Additonal file 1: Table S-T6). Abbreviations for sensory descriptors: *PW *= *PatternWhite*, *PB *= *PatternBlack*, *Co *= *Contrast*, *Cu *= *Curls*, *Re *= *Regular*, *MS *= *MultiShade*. (see Additonal file 1: Table S-T5). Higher-order interaction terms: *pI *= [*θ*_D_-], *pII *= [*θ*_D _+, *θ*_N_+, *p*_D _≠ *p*_N_], *pIII *= [*θ*_D_+, *θ*_N_-, *p*_D_+].

The parameter ranges found to be of interest in the preliminary exploration (1) (see Additonal file 1: Table S-T2) were used for designing the subsequent two experiments (2) and (3). The image analysis filters employed in the extensive screening experiment (2) (see Additonal file 1: Table S-T3) revealed systematic co-variation patterns in the image analysis profile data. But these patterns were difficult to interpret. The main, sensory-based experiment (3) employed a modified version of the reduced design (see Additonal file 1: Table S-T4), and consisted in using a trained sensory panel to define verbal pattern descriptors and then quantify the patterns in each image in terms of these descriptors. Based on input from the investigators the sensory panel defined 12 descriptors which they considered sufficient and by which they subsequently quantified the patterns (see Additonal file 1: Table S-T5). This allowed us to develop a PD model (Figure 4) by bi-linear PLSR regression in latent variables, relating the chosen 12 sensory descriptors (see Additonal file 1: Table S-T5), to the seven model parameters via 3 latent variables, over the 32 images. Perturbations in the model parameters *θ*_D_, *θ*_N_, p_D _and p_N_, alone and in combinations, clearly affected the solutions in systematic ways, while variations in *μ *(the ratio between the degradation rates of Delta and Notch) and in the initial conditions had fairly small effects. Based on split-half cross-validation [12], the PD model correctly predicted 88% of the variance in the 12 sensory descriptors from the model parameters. Conversely, 85% of the variance in the selected non-linear model parameter combinations was correctly predicted from the pattern descriptors.

We found that the PD model clustered the 32 graphical patterns in four main classes, here named I, II, III, IV (see Additonal file 1: Table S-T6). In a cross-validation experiment in which each image was treated as unknown against a PLS discriminant analysis model estimated from the other 31 images, the set of pattern descriptors could be used for successful classification of each of the 32 images into the four classes. The clustering was confirmed by independent hierarchical cluster analysis of the patterns based on their Euclidean distances in PD space, and appears to be quite robust (see Additonal file 1: Figure S-F3).

Images belonging to one and the same class share obvious visual pattern characteristics that distinguish them from images from the other classes, and can be associated with different domains in parameter space (see Additonal file 1: Table S-T6, Figure S-F4). For example, low values of *θ*_D _are a clear indication that the resulting pattern will fall into class I. The images in class I are likely to be high in the descriptors *Sharpness*, *Contrast *and *PatternBlack*, and low in *Whiteness *and *PatternWhite*, implying primarily black and white cells, the former outnumbering the latter and participating in the curls which dominate the pattern in this class (Figure 2A).

### Parameter-dependent differentiation phenomena

We subsequently (Phase 4) checked that the clustering is not just an artefact stemming from the low number of patterns in the fractional factorial design by running the fourth and final set of simulations to pursue details. We varied the Delta threshold parameter *θ*_D _in small steps to traverse the PD space along a straight line, illustrated in the biplot of Figure 4a from the previous simulation experiment, from class I (*θ*_D _= 0.1, characterised e.g. by sensory descriptor *Sharpness*) via class III (*θ*_D _= 0.7, characterised by *Whiteness *and *MultiShade*) and up to *θ*_D _= 0.9. Some new sensory descriptors were added (see Additonal file 1: Table S-T7).

**Figure 5:**
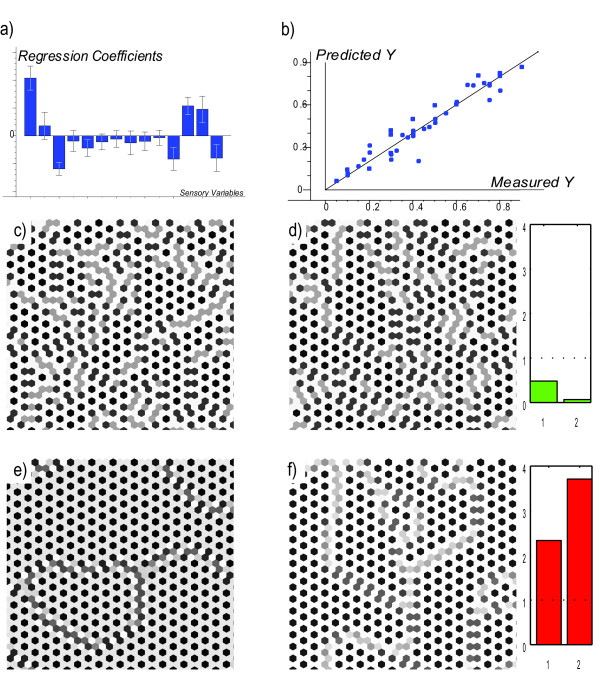
**Predicting model parameters from solution characteristics**. Top row: multivariate calibration illustrated. (a) Regression coefficient for predicting *θ*_D _from sensory profiles, estimated by PLSR. While *θ*_N _was kept constant at 0.1, *p*_D _and *p*_N _were likewise calibrated for. (b) Predicted *θ*_D _*vs*. known *θ*_D _in the calibration samples, based on cross-validation to avoid over-fitting. Middle row: prediction in a normal sample. (c) A solution image generated with *P *= [*θ*_D_, *θ*_D_, *p*_D_, *p*_N_] = [0.7, 0.1, 10, 3]. (d) Image reconstructed from predicted sensory parameters *P *= [0.71, 0.1, 9.43, 3.57]. Right: outlier tests (1, relative leverage; 2, residual variance) indicate a valid prediction. Bottom row: prediction in an abnormal sample. (e) An image generated with a parameter combination outside the range calibrated for, *P *= [0.7, 0.5, 10, 3]. (f) Image reconstructed from predicted sensory parameters *P *= [0.79, 0.1, 11.3, 1.7]. The reconstruction is obviously bad, as expected. But since outlier tests (right) were too high, the invalid prediction caused automatic outlier warning.

**Figure 6:**
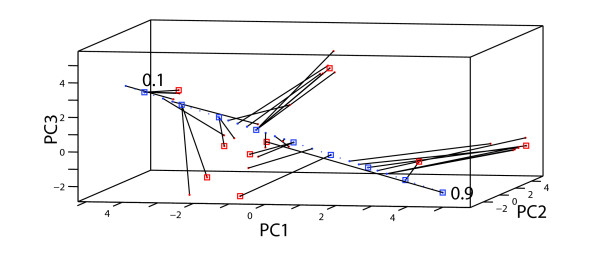
**The sensory appearance of solution images jumps between distinct regions in the property space spanned by the three first principal components when *θ*_D _increases from 0.1 to 0.9**. Each of the 34 line segments connects the sensory properties of each image to its *θ*_D _value. A subset of 9 solutions, simulated over a 51 × 51 lattice with fixed set of initial states, equally spaced at *θ*_D _= 0.1, 0.2,..., 0.9 are marked by red and blue squares, respectively. Parameters kept constant: *θ*_N _= 0.1, *p*_D _= 10, *p*_N _= 3.

From these results, good predictive PLS regression models were developed between the sensory descriptive profile and the model parameters within the parameter range calibrated for, as exemplified for *θ*_D _in Figure 5. Moreover, rather than spreading evenly in PD space, the image properties jump between a few clearly separated domains in the 3-dimensional PLS component space, with few intermediates (Figure 6).

**Figure 7:**
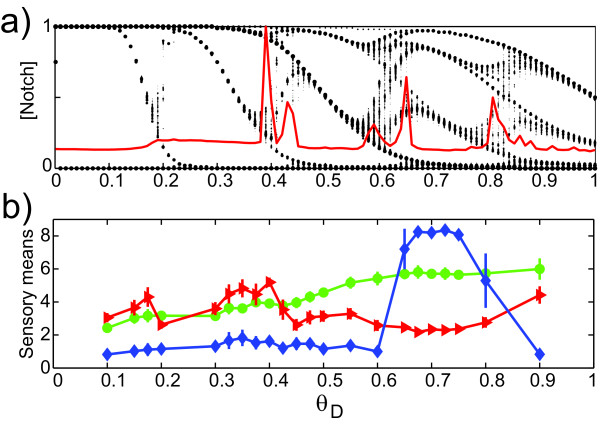
**(a) The Notch differentiation pattern obtained for 0 <*θ*_D _< 1 in the 51 × 51 lattice with periodic boundary conditions**. Patterns with *θ*_D _≅ 0.1 belong to Class I, with *θ*_D _≈ 0.7 to Class III. The superimposed solid curve shows the relative convergence time required to reach the stable state. Other parameter values: *θ*_N _= 0.1, *p*_D _= 10, *p*_N _= 3, *μ *= 0.5, *M *= 1, and *s *= 0.2. (b) Weighted averages of the ratings of *Whiteness *(green), *ThicknessCurls *(red), and *Twoheadedness *(blue) by the sensory panel *vs. θ*_D _in the 51 × 51 lattice with periodic boundary conditions (see Additonal file 1: Table S-T7). Vertical bars indicate standard deviations. The broad maximum for *Twoheadedness *corresponds to class III.

As *θ*_D _increased, the estimated Notch activity passed through a series of complicated, abrupt changes resembling bifurcations (Figure 7a), showing that the statement of Collier *et al*. [7] that the patterns are insensitive to the precise values of the model parameters is only partially true. Ordinary bifurcation diagrams show the number of coexisting stable states of which the system may occupy just one at a time, but Figure 7a shows the number of stable Notch levels actually expressed simultaneously over the lattice for each value of *θ*_D_. That is why this is not a bifurcation diagram in the common sense. We propose to call it a *differentiation diagram*, where *θ*_D _is a *differentiation parameter *regulating the number of simultaneous levels, or the degree of differentiation. This being said, the diagram shows a large number of "bifurcation" values of *θ*_D _for which the number of simultaneous Notch levels changes. The number of levels is related to the complexity of the image: a high number of levels come with a complex and highly irregular pattern. A high number of equilibrium levels, e.g. at either side of *θ*_D _= 0.41 and 0.62, correlates with long integration time (Figure 7a). Both can be seen as a consequence of high *frustration *[9], which implies that the system needs extensive fine-tuning and either takes a long time to settle in a stable, patterned state or is chaotic. Despite the fact that no descriptor was designed with analysis of differentiation in mind, the descriptors characterise the patterns in a way that at least partly reflects the complicated differentiation pattern for varying *θ*_D _(Figure 7b).

### Initial state variations, repeatability and boundary effects

For a given set of parameter values the total number of stable states in a large network is probably very large. However, each initial state (*N*^0^, *D*^0^) lies in the attractor basin of just one patterned state. An important question is whether the patterns generated by all (*N*^0^, *D*^0^) for a given set of parameter values lead to more or less the same state in PD space. For a realistic model of a biological system we would expect this to be true, as biological systems are almost by definition structurally stable and thus insensitive to internal and external stochastic fluctuations. Thus, contrary to stable points in phase space, we would expect points in PD space to have large attractor basins.

Once the clustering into four classes was discovered during the multivariate analysis of the sensory data, it was straightforward to test the robustness and the visual appearance of these pattern types. Varying initial conditions within the imposed limits (see Methods) changed the details of the patterns without appreciably altering their descriptor profiles. This is illustrated by the proximity of the two independent random initial condition replicates in class IV (Figure 4a, open triangles Δ, ▽). The precision of the sensory descriptive analysis is also illustrated in the same figure by the closeness of the three independent sensory parallels within each of these two initialisation replicates.

With periodic boundary conditions, which are commonly used to mimic an infinite domain, we expected that the boundary effects would be negligible as regards the pattern characteristics. This was confirmed by simulations in which the original 50 × 50 lattice was embedded in a larger (75 × 75) lattice which was run with the same set of random initial perturbations as in the 50 × 50 lattice. The final patterns in the 50 × 50 lattice in the two cases belonged to the same class, and in most cases were almost identical (data not shown). Some very large-scale spatial patterns appear as boundaries between domains with a three-periodic pattern, but with a phase shift along the boundaries (Figure 3B). In an infinite-sized lattice we expect these patterns would fail to appear. By increasing the pattern size to 51 × 51 we found that some of these changed to a 3-periodic pattern all over the lattice without phase-shift boundaries (51 is divisible by 3).

## Discussion

Spatial patterns are determined by the concentrations of certain species in each separate cell. But to handle and analyse differentiation and patterning we need mathematical variables that express the macroscopic, essential properties that allow us to describe, classify, and distinguish qualitatively different patterns in biologically meaningful ways. To this end it is of little value to specify the equilibrium values and positions of millions of individual cells. The macroscopic pattern is an emergent, aggregate property, and the pattern characteristics of interest can only be described meaningfully by new, dedicated variables defined explicitly to describe and quantify just the macroscopic properties of the patterns that the modellers deem of interest.

For a moderately sized model, any number of numerical simulations can be run and almost unlimited amounts of simulation data can be stored with modern high speed and high capacity computers. However, more data does not necessarily lead to more insight. The challenge is to maximise discovery, overview and statistical power, while minimising work and risk of over-interpretation. Multivariate data modelling in latent variables has proven to be a highly useful approach for extracting and displaying the main co-variations in high-dimensional response profiles.

In the present case, we chose a pragmatic explorative approach, involving qualitative methods that facilitate the detection and conceptualisation of novel phenomena, and quantitative methods that permit modelling, prediction, validation and interpretation. For each stage in our approach there exist general and well-documented methods that have proved their efficiency and reliability in a large number of applications. As others have experienced before us in various settings, the human visual system combined with a task-specific vocabulary is a scientifically reliable and superbly versatile tool [15] for finding, quantifying and communicating regular as well as irregular patterns. For example, we discovered the pattern type "two-headedness" (short, crooked "worms" with two black "heads" connected by a gray "body" on a white background, Figure 2D) because the Class-III parameter combination showed abnormally high scores of the sensory descriptor *MultiShade *in the 50 × 50 lattice in the first sensory experiment. Once this feature was discovered, we developed a specific term, "Two-headedness" for it, with the descriptor *Twoheadedness*. The sensory panellists were "calibrated" (trained on reference images) for this trait to ensure intersubjectivity and repeatability. Then it was quantified along with the other sensory descriptors in the second sensory experiment (Figure 5), which allowed it to be compared to the differentiation diagram (Figure 7). Prior to its detection, it was difficult to pick up this strange feature by the automatic computerised image analysis. But after having discovered it during the sensory assessments we were able to construct a specific mathematical image analysis filter that quantifies it. This will be described elsewhere.

Our set of descriptors is clearly not sufficient to describe or classify all images generated by the model. That was never our goal. We selected those descriptors that we found best suited to describe those pattern features that after the initial scans appeared to be most interesting to us. Models are always constructed with a purpose, and the researcher's interest will in general be focused on those aspects of the images that are relevant for this purpose. A need for extensive investigations of complex model arises simply because one cannot say in advance how the model will behave and which behaviours may turn out to be of interest and importance.

It seems that our approach promotes effective investigation of the outcomes of pattern-generating models (and possibly of complex mathematical models in general), and is characterised by a number of attractive features. It is *cost-effective*: initially screening many conditions by low-cost, but non-specific profiling test methods, and then pursuing selected conditions by more informative methods. It is *statistically stable*: only relationships that show predictive ability in "secret" simulation (cross-validation) are accepted. It is *focused*: searching for a description of only those properties of the original model that are of interest, ignoring all irrelevant details and leading to an enormous phase space reduction. It is *wide-ranging*: yielding an overview as well as the ability to predict details. It is *analytic*: providing an explanation of the dominant pattern features in terms of properties of the original model, i.e. of the real system that is being modelled. It is *adaptive*: if the researchers' attention should be turned towards other or additional features, the model can be adapted or replaced by another system-level model generated in the same fashion. It is *inter-subjective*: using standardised and well-tested methods from sensory science and chemometrics helps to keep subjectivity due to the researchers' individual bias and preferences to a minimum. It leads to a *system level model*: seeking a description and understanding of the emergent phenomena and features of the patterns that are the result of the system as a whole and that only have meaning on the level of the system itself, and relating the relevant characteristics of the final patterns directly to the system parameters without specifying the levels of every single cell. As far as the relevant features of the final, stable patterns are concerned, the system-level model replaces the original ODE-based pattern-generating model. Given a set of parameter values, we can predict the corresponding type of pattern without actually integrating the system, and given a pattern, we can predict the parameter domains leading to it.

In our opinion, there is little hope that realistic multi-scale spatiotemporal cGP models which explain high-dimensional phenotypic variation associated with the emergence of biological structure, organisation and function through differentiation, can be expressed in a simple way. In contrast to our simple illustration model, realistic pattern-generating cGP models will in general be dynamic, non-linear and complex, with numerous variables and parameters, possessing a hierarchical structure and perhaps containing continuous processes as well as discrete event processes, and discrete or distributed time delays. They will also have to deal with noise and stochastisity as well as random perturbations during the course of development. Large and complex models for open systems will possess emergent features that cannot be inferred from the building blocks of the model, and may generate a large repertoire of different mathematical behaviours depending on initial values and boundary conditions, parameter values and details of the functional relationships. In this connection the behaviour of the model could simply mean its ensemble of final, stable states, or the transient motions leading to these states might also be included. In either case, achieving a complete survey of all possible, relevant behaviours is hardly possible using a traditional mathematical analysis where analytical investigations are combined with intuition-based simulations and global or individual parameter fits to data. That approach is widely used, but may fall short of disclosing the full range of possible behaviours of even quite simple models. As the disclosure of a substantial part of the phenotype space is a prerequisite for developing comprehensive genotype-phenotype maps, there is indeed a need to develop robust and generic methodological standards for discovering the behavioural repertoire of complex patterning models. It is likely that combined with analytical investigations and numerical "experiments", a factorial design approach as outlined here will become part of such a standard.

## Conclusion

The number of cells could have been increased many-fold without complicating the analysis apart from being computationally more demanding. This suggests that our approach could be used on biologically more relevant and mathematically more complex pattern models with many chemical species, larger numbers of parameters and more complex interactions. Scaling issues may appear, though, and we do not know how human pattern description scales up to three dimensions and irregular cell lattices. This should be addressed so that we can make a realistic assessment of to which degree a combined use of system dynamics methodology and multivariate data modelling in latent variables has the potential to become a generic approach to explain higher-order differentiation phenomena.

## Methods

### Model analysis

With suitable boundary conditions, for example periodic, the model in Eqs. (1) has a homogeneous equilibrium (D*, N*) given by the unique solution of D* = 1-*S*(N*, θ_N_, *p*_N_), N* = *S*(D*, θ_D_, *p*_D_). This state is unstable unless the steepness of the sigmoid functions is very gentle [7,17], and a perturbation of the homogeneous state will initiate a transient during which cells develop towards different final states, creating a spatial pattern. It is far from obvious which parameter value combinations generate which patterns and how patterns could be described and classified.

The patterns were obtained by integrating Eqs. (1) numerically from initial values *D*_*k*_^0^, *N*_*k*_^0 ^on a 50 × 50 or 51 × 51 lattice of hexagonal cells with periodic boundary conditions. The lattice dimensions 51 × 51 were chosen to avoid certain shifts in the basic three-periodicity entirely due to the periodic boundary conditions (50 is not divisible by 3). The initial values for the individual cells represented small, random perturbations from the homogeneous steady state value D*. To generate clearly different initial value sets, we modified the maximum size *M *and sign *s *of the perturbations (see Additonal file 1: Table S-T1), if *s *> 0 (< 0), all perturbed values were larger (smaller) than the equilibrium value. Thus, including our parameterisation of the initial values, our model has a total of seven parameters which span the Parameter Space.

### Simulations and descriptive analysis of patterns

As a start we make some useful distinctions. For each choice of parameter values and initial values the model yields a stable solution, from which we generate a two-dimensional *image *in which each cell is given a shade of gray defined by its Notch level (white: *N *= 0, black: *N *= 1). The image exhibits certain pattern characteristics, in short, a certain pattern. A pattern is a conspicuous, emergent feature of an image (could be periodic or non-periodic). The pattern characteristics, e.g. Whiteness, are qualities. If the overall Notch level is low, we characterise the image by attaching the quality Whiteness to it. Using sensory methods or dedicated mathematical filters, the degree of whiteness is given by the value of the scalar variable *Whiteness *for this particular image.

The most interesting ranges for the individual model parameters were sought by trial-and-error in an initial exploration (1). Each parameter combination was tested with a new set of random perturbations for the simulation. A second, full factorial screening design 2^7 ^with centre-points (2) was then chosen as a compromise between computational load and probability of finding important patterns (see Additonal file 1: Figure S-F1, Table S-T2). The multivariate profiling (see Additonal file 1: Table S-T3) and data analysis (cross-validated PLS regression of the relationships between the design parameters and about 80 automatically generated image analysis descriptors) showed that several kinds of pattern were indeed generated by the simulations (see Additonal file 1: Figure S-F2). Based on preliminary results, the design was expanded in a couple of stages to ensure that the relevant parameter domains were adequately spanned.

To ensure a more informative and interpretable characterisation, we decided to apply human visual evaluation of an interesting subset of the solutions in the main experiment (3). Since this is a more laborious and expensive measuring principle, a reduced statistical design was chosen for this third stage. From the original screening design, a fractional factorial design 2^7-2 ^was selected by combining a high or low value of each of the seven parameters, confounding the least interesting main effects with higher-order interactions of other factors (see Additonal file 1: Table S-T4). Six of the 32 chosen parameter combinations resulted in a return to the homogeneous equilibrium, and were replaced by three centre points in two replicates with different initial conditions.

For each of the 32 experimental conditions, the full 50 × 50 equilibrium lattice of Notch was printed on paper in black-and-white. The sensory descriptive analysis, performed by a panel of eleven assessors working as trained sensory judges at the Norwegian Food Research Institute, had two distinct steps, carried out according to the ISO method convention [13], as e.g. used in [14]. (i) The assessors and the researchers agreed on twelve descriptors reflecting characteristic pattern features (see Additonal file 1: Table S-T5). The assessors were then trained to respond similarly with respect to each descriptor. (ii) Each image was judged with respect to each of the twelve descriptors on a scale from 1 to 9 by the eleven individual assessors, with the images anonymised and presented in random order. Finally, PLSR with split-half cross-validation was used in various ways to find reproducible patterns of co-variation between the twelve sensory descriptors and the seven design factors and their interactions (Figure 4). Graphical inspection of the PLS score plot (Figure 4a) as well as cluster analysis led us to group the solutions into four distinct classes (see Additonal file 1: Figure S-F3, Table S-T6).

In order to pursue in detail the effect of changing the parameter *θ*_D _from the low values giving class I through class III (*θ*_D _≈ 0.7) up to *θ*_D _= 0.9, a final sensory analysis experiment (4) was performed on simulation results for 36 values of *θ*_D _and constant values of the other parameters, this time using a 51 × 51 lattice and a fixed set of randomly chosen initial values (Figure 7). To focus on the local fine-structure of the images, the panel and the project leaders agreed upon a slightly revised set of twelve descriptors of which two are reported here (see Additonal file 1: Table S-T7). The eleven assessors and the general evaluation conditions were the same as before.

## Authors' contributions

HM, SWO and EP developed the original idea, SRV and HM planned the numerical experiments, SRV and DB created the computer image analysts descriptors, MM was responsible for the sensory analysis, SRV and HM performed the multivariate statistical analyses, EP edited the manuscript, all authors contributed to and approved the final manuscript.

## Supplementary Material

Additional file 1 **The file contains a more detailed presentation of the method, including parameters and parameter values, extracted pattern features, sensory descriptors, and pattern characterisations.** Various graphs and diagrams illustrate and present results from the PLS analysis. Click here for file

## References

[B1] Rajasingh H, Gjuvsland AB, Vage DI, Omholt SW (2008). When parameters in dynamic models become phenotypes: A case study on flesh pigmentation in the chinook salmon (*Oncorhynchus tshawytscha*). Genetics.

[B2] Ehebauer M, Hayward P, Arias AM (2006). Notch, a Universal Arbiter of Cell Fate Decisions. Science.

[B3] Le Borgne R (2006). Regulation of Notch signalling by endocytosis and endosomal sorting. Current Opinion in Cell Biology.

[B4] Louvi A, Artavanis-Tsakonas S (2006). Notch signalling in vertebrate neural development. Nature Reviews Neuroscience.

[B5] Massague J (1990). Transforming growth factor-alpha - a model for membrane-anchored growth-factors. J Biol Chem.

[B6] Meir E, von Dassow G, Munro E, Odell GM (2002). Robustness, Flexibility, and the Role of Lateral Inhibition in the Neurogenic Network. Current Biology.

[B7] Collier JR, Monk NAM, Maini PK, Lewis JH (1996). Pattern formation by lateral inhibition with feedback: A mathematical model of Delta-Notch intercellular signalling. Journal of Theoretical Biology.

[B8] Podgorski G, Bansal M, Flann N (2007). Regular mosaic pattern development: A study of the interplay between lateral inhibition, apoptosis and differential adhesion. Theoretical Biology and Medical Modelling.

[B9] Binder PM (2008). Frustration in Complexity. Science.

[B10] Wold S, Martens H, Wold H, Dold A, Eckmann B (1983). The multivariate calibration problem in chemistry solved by the PLS method. Matrix Pencils.

[B11] Martens H, Næs T (1989). Multivariate calibration.

[B12] Martens H, Martens M (2001). Multivariate analysis of quality: an introduction.

[B13] ISO (2003). Sensory analysis Methodology General guidance for establishing a sensory profile.

[B14] Martens H, Thybo AK, Andersen HJ, Karlsson AH, Donstrup S, Stodkilde-Jorgensen H, Martens M (2002). Sensory analysis for magnetic resonance-image analysis: Using human perception and cognition to segment and assess the interior of potatoes. Lebensmittel-Wissenschaft Und-Technologie-Food Science and Technology.

[B15] Meilgaard M, Civille GV, Carr BT (1999). Sensory evaluation techniques.

[B16] Martens M, Veflingstad SR, Plahte E, Bertrand D, Martens H A sensory scientific approach to visual pattern recognition of complex biological systems. The 8th Pangborn Sensory Science Symposium, 26-30 July 2009, Florence, Italy.

[B17] Plahte E (2001). Pattern formation in discrete cell lattices. Journal of Mathematical Biology.

