# Supplementary material for: The genotype-phenotype relationship in multicellular pattern-generating models - the neglected role of pattern descriptors
Source: BMC Syst Biol. 2009 Sep 4;3:87. doi: 10.1186/1752-0509-3-87 (PMC2749810; doi:10.1186/1752-0509-3-87)
Supplement: Additional file 1 — The file contains a more detailed presentation of the method, including parameters and parameter values, extracted pattern features, sensory descriptors, and pattern characterisations. Various graphs and diagrams illustrate and present results from the PLS analysis. [file 1752-0509-3-87-S1.pdf]

Supporting material for

Harald Martens, Siren R. Veflingstad, Erik Plahte, Magni Martens, Dominique Bertrand, Stig W. Omholt *BMC Systems Biology* 2009, **3**:87

The genotype-phenotype relationship in multicellular pattern-generating models – the neglected role of pattern descriptors

Corresponding author:

harald.martens@umb.no

Details and explanations, mainly based on

Siren Røst Veflingstad (2006) The search for relations between structure and behaviour in models of gene regulatory networks. PhD Thesis, Norwegian University of Life Sciences / CIGENE.

More details will be given in

Martens, M., Veflingstad, S.R., Plahte, E., Bertrand, D., and Martens, H. (2009) A sensory scientific approach to visual pattern recognition of complex biological systems. Oral presentation 8th Pangborn Sensory Science Symposium 26-30 July 2009, Florence, Italy, www.pangborn2009.com. (paper in prep.)

and

Isaeva, J., Sæbø S., Wyller J., Liland, K.H., Færgestad E.M, Bro, R. and Martens H (2009) Using GEMANOVA to explore the properties of dynamical systems models (working title)

Content:

1. Introduction

2. Explorative screening experiments

2.1 Preliminary exploration: Getting to know the system

2.2 Extensive screening

2.2.1 Spanning the parameter space by full-factorial designs

2.2.2 Characterizing the solutions by mathematical profiling

2.2.3 Exploring the results by multivariate soft modelling

2.2.4 Conclusions from the explorative screening experiment

3. Sensory descriptive experiment

3.1 Spanning the parameter space by reduced factorial design

3.2 Characterizing the solutions by human sensory profiling

3.3 Exploring the results by multivariate soft modelling

3.4 Conclusions from the sensory descriptive experiment

4. Pursuit of discovered details

4.1 Spanning the parameter space by dense sampling

4.2 Characterizing the solutions by human sensory profiling

4.3 Exploring the results by summary histograms and graphics

4.4 Conclusions

5. Tables and figures

1. Introduction

The research article presents and illustrates a new, generic approach to top-down explorative analysis of a complex mathematical model. The main text outlines the approach, organized according to conceptual topics and main results. Considering the wide span of research cultures combined in this kind of work, the condensed printed form is here supplemented with more detailed documentation, following the actual structured discovery process step-by-step.

Experiences from explorative multivariate data modelling in agronomy, food research and functional genomics were applied in order to study a chosen set of differential equations constituting a cell differentiation model: This theory-driven "hard" mathematical model (i.e. a concise mathematical description representing the equivalent of a "hard-wired" version of the scientists' assumptions and choices) was treated as if it were a real-world complex object type, e.g. a barley variety or a yeast strain: It was perturbed systematically in various ways, "grown" (integrated to steady state in the computer) , and described in many different ways. The ensuing data tables were then interpreted by data-driven "soft" multivariate data modelling (i.e. a mathematical approximation models using as few prior assumptions and choices as possible, and displaying the main patterns of co-variations in the data). Based on the results in the project reported, a new research project has recently been initiated.

All the computations were done in either Matlab™ (www.mathworks.com), using the authors' code, or in The Unscrambler (www.camo.com).

2. Explorative screening experiments

Figure 2 in the main publication outlines the four consecutive steps in the explorative investigation of a high-dimensional non-linear dynamic model, from overwhelmed bewilderment via three cycles of exploratory induction and confirmatory deduction, to final discovery of unexpected pattern types and interpretation of the differentiation complexities. Here is an outline of the four stages:

2.1 Preliminary exploration: Getting to know the system

This first research cycle consisted in trying to get a first overview of the main types of patterning that the dynamic model is capable of, and to identify which parameters and parameter- combinations seem to be most important. The system has five model parameters and two parameters controlling the random initialization. For each of them, an upper and a lower level were chosen, based on trial-and-error.

2.2 Extensive screening

The main ambition is now to obtain the mapping from parameter space to solution space. This is achieved by numerical simulation of the model using a number of different parameter combinations.

2.2.1 Spanning the parameter space by full-factorial designs

In the second cycle, a high number of computer simulations are performed according to a suitable *experimental designs* to ensure that the parameter space is spanned systematically in the regions found in the first research cycle to be of interest. The factorial designs allow identification of the effects of different parameters separately and in combination. An efficient design for studying the joint effects of multiple factors on different responses is a factorial design in which all possible combinations of the levels of the different factors are investigated. In the case of many factors with multiple levels, the number of combinations quickly becomes very large and an alternative is to perform an experimental design in two steps: (1) Set up and employ screening designs in which each factor has two levels, a so-called *2^k factorial design*, where *k* is the number of factors. With only two levels we assume that the response is approximately linear over the range of the factors chosen. By including a centre point and axial points (Fig. S-F1), we also get a chance of detecting non-linear parameter-solution relationships, although at low resolution. Since low-cost evaluation methods were employed at this stage, we could afford to use several such designs in succession, in adjacent regions of the parameter space; only some of them will be reported here. (2) The screening design may be extended in the most interesting factors and directions. The remaining factors are varied according to the original two-factorial design including centre and axial points.

Of the seven factors defined in Table S-T1, 1-6 are continuous. For these we get e.g. a 2^6 factorial design in addition to a centre point and the axial points, as illustrated in (Fig. S-F1) for three such design factors. The last factor, *PertDir* is a category variable, and the notion of a centre value does not apply. Table S-T1 describes the parameters that were systematically varied, along with the levels chosen. This resulted in a total of about 150 different simulation conditions.

There are other parameters that may influence the behaviour of the system, but which have been kept constant in this study. First of all, all cells in the lattice are treated equally. Thus, there is no variation in the model parameters among the different cells, with one exception. When all cells are perturbed, the direction of perturbation is equal for all cells, but the magnitude must vary within the range given by *PertSize* because the homogeneous state is stable under homogeneous perturbations. Second, the perturbation always takes place in the variable *D*. And finally, we have assumed periodic boundary conditions, mimicking an infinite domain with a finite number of cells. Table S-T1 also outlines the modelling aspects that were kept constant.

Initial analyses showed that the sigmoid threshold values appeared to be the most important parameters in determining the behavior of the system, and we therefore expanded the design in these two parameters (θ_D and θ_N). The extension consists of increasing the low and high value of the initial design for both thresholds separately as well as simultaneously (Table S-T2). The remaining factors are not changed. The extended design resulted in a total of about 600 different simulation conditions, each of which was data-analytically regarded as a separate "sample".

A note on notation: A *sample* corresponds to the solution of the model in Eq. (1a,b) obtained with a *set of parameter combinations* from the experimental design. For the feature extraction, we also generate an *image* for each sample. (see e.g. Fig. 2).

Initially, the experimental design resulted in 616 samples to be submitted to analysis. However, there were two issues that reduced this set: stability of the homogenous

steady state and problems with convergence. There were 152 parameter combinations resulting in a stable, *unpatterened* steady state. As these systems clearly have a final state very different from the systems that destabilise and move towards a *patterned* state, they were also removed from the set before analysis.

With respect to convergence, there were sets that resulted in extremely slow convergence (if convergence at all) to a patterned state. Thus, for the simulations, we set an upper limit for the time of simulation, implying that if convergence to a patterned state was not reached within this limit, the simulation was stopped. The systems whose simulation was stopped because the time limit had been reached, might not have attained their true patterned state, and were therefore not included in the analysis. A total of 85 parameter combinations did not converge to a steady state within reasonable computer time; these are also ignored in the following.

2.2.2 Characterizing the solutions automatically by mathematical profiling

We are interested in finding relations between chosen model parameters and the resulting patterns in the final heterogenous states (given that they exist). As we initially only have superficial knowledge of what these patterns may be, we need general methods for characterising the final states of the different samples, with little cost and effort. An overview of the features and their description is given in Table S-T3. Below follows a general description of the three main types of features.

Greyscale

First of all, the final state of the lattice was characterised by its distribution of values of Notch (*N)* and Delta (*D)*. In the final states, the value of *N* (or *D*) determines the darkness of a cell (for an example, see images in Fig. 2, picturing the level of *N* in each cell), the extremes being white for *N* = zero and black for *N* = 1. Thus the indexes associated with the final distribution describe features as whiteness (or blackness) as well as contrast or homogeneity.

Spatial autocorrelation

The features for greyscale do not contain any spatial information. Spatial autocorrelation, on the other hand, only contains spatial information, mainly in terms of periodicity. Spatial autocorrelation is defined by

(S-E1) $$A_{(m,n)}(\mathbf{X}) = \frac{\sum_{i,j}[\mathbf{X}(i,j)-\overline{\mathbf{X}}][\mathbf{X}(i+m,j+n)-\overline{\mathbf{X}}]}{\sum_{i,j}[\mathbf{X}(i,j)-\overline{\mathbf{X}}]^2},$$

where $\overline{\mathbf{X}}$ is the mean of matrix **X**, and (*m*,*n*) corresponds to a shift of **X** by *m* rows and *n* columns. For this specific system, we let $0 \le m,n \le 25$, and computed *A* using the final levels of *N*.

The linear analysis of Collier *et al.* predicted a period-3 pattern to be dominant. Thus, we defined features in order to check for this periodicity specifically (*ALp3corr*, *AGp3corr* and *ADp3corr* in Table S-T3). This was done by comparing the computed

autocorrelation matrix with the corresponding matrix obtained for a perfect 3-periodic pattern.

Cluster analysis of objects

A pattern is clearly a combination of level and spatial information. Thus, in addition to indexes describing each of these separately, we derived a set of indexes combining the two (object analysis, Table S-T3). These indexes are based on the detection and characterisation of connected regions, that is, regions with same pixel values, in an image. In the following, these connected regions are denoted *objects*. The derivation of these indexes was implemented in the following way.

First, a tiff-image of each sample was generated based on the level of N in each cell. The levels of N were discretised to four levels of grey ($0 < N \leq 0.25$ equals white, $0.25 < N \leq 0.5$ equals light grey, $0.5 < N \leq 0.75$ equals dark grey, and $0.75 < N \leq 1$ equals black) before generating the image. The image was then analysed for each of the four levels separately, by setting the value of each cell within the given grey level under study equal to one and all other cells equal to zero. We then detected objects in the resulting binary image and characterised them with respect to different properties (Image Analysis Toolbox, Matlab®). It turned out that this either returned very many objects or only one object when analysing each of the grey levels. Thus, we applied the morphological operations *dilation* and *erosion* to enhance the interesting structures in the image. Dilation expands the objects by adding pixels to the boundaries of objects, while erosion removes pixels on object boundaries, thus reducing the objects. For each of the four grey levels we generated four filters of the image: the original, a dilated, an eroded, and finally, an image that was first eroded and then dilated. Thus, in total, 16 versions of each sample (4 grey levels times 4 morphological filters) were analysed.

In all 16 versions, the objects were characterised with respect to a set of 11 properties (area, perimeter, shape etc.), and these characteristics provided the basis for a cluster analysis of the objects using the K-means approach. The cluster analysis was applied simultaneously on *all* objects in *all* filters in *all* images. Some filters only returned one object (the whole lattice), and this object was removed from the set of objects prior to cluster analysis, and treated separately (see below). The result of the cluster analysis was the $N_{\text{object}} \times N_{\text{cluster}}$ matrix **C,** where element c_{ij} is 1 if object i belongs to cluster j and zero otherwise. Each object may only belong to one cluster. A measure of the relative dominance of a given cluster was obtained by multiplying each column of **C** by the average area of all objects in that cluster. In order to summarise the cluster information for each *image* (and not for each object, as it is in matrix **C**), we first found the average and maximum of all objects in a given filter (dilated, eroded; white, black and so on). Then we computed the average and maximum of all filters belonging to an image, resulting in the matrix of size $N_{\text{sample}} \times 2N_{\text{cluster}}$, where the first N_{cluster} columns denote the average and the remaining N_{cluster} columns denote the maximum (indexes *CiMean* and *CiMax*, respectively in Table 4, where i denotes cluster number). In order to handle the filters returning only one object, we defined the 16×16 diagonal matrix **E** for each sample. Here each element e_{ii} is 1 if filter i contains one object and zero otherwise. All other elements ($e_{ij}, j \neq i$) are zero. A filter with only one object implies that there are either no cells with the given level of grey or that this level of grey is so dominant in the lattice that the whole lattice is returned as a single object. Finally, the mean was taken over all the filters of an image,

returning a $N_{\text{sample}} \times N_{\text{filter}}$ matrix with the features *CWRaw*, *CWDil*, *CWErod* and so on, as stated in Table 4.

It should be mentioned here that there are numerous other methods for extracting features of images (for example, wavelet analysis, angular measurement technique, singular value decomposition). We have tested some of them, but have found that for the current system, the indexes described in Table S-T3 for our purpose provide the best descriptors, partially in terms of explaining the patterns and partially in terms of interpretability. To summarize, the final data set consisted of the following set of matrices for each of the perturbation types: (i) the matrix of design factors (parameters and initial conditions; $N_{\text{sample}} \times 7$), (ii) the matrix of characteristic features ($N_{\text{sample}} \times 78$). The latter matrix consists of three submatrices, one each for each of the classes of features (Table S-T3).

2.2.3 Exploring the results by multivariate soft modelling

The data were analysed by partial least squares regression (PLSR), as implemented in The Unscrambler® (www.camo.com). Two different, but related PLSR approaches were applied. An ANOVA-like PLSR (APLSR) estimates the effects of the design factors on the extracted features. In other words, the **X** is the matrix of design factors while **Y** is the matrix with values for the characteristic features. The converse, called a PLSR discriminant analysis (PLS-DA), seeks to explain the design factors from the characteristic features of the system. All input variables were standardized (i.e., their value was divided with the standard deviation of the respective feature) prior to analysis. The number of optimal principal components was in both analyses determined by cross-validation, while the reliability of the regression coefficients was correspondingly estimated using jack-knifing.

Before presenting the analysis for all the features, a comment on the local pattern is at its place. As mentioned above, we defined several indexes describing deviation from a perfect 3-periodic pattern. The data show that on a local scale (that is, only looking at the autocorrelation within 3 cells), there are several samples that have a pattern that is very close to this 3-periodic pattern, but the majority of the samples have relatively large deviations (see e.g. Fig. 2). Looking at the same property for the full autocorrelation matrix, all samples deviate considerably. Thus, there are clearly other structures present in the lattices, which the linear analysis of Collier *et al.* is not able to predict.

To summarize the "soft modeling " of the simulation results, the correlation loadings for the PLS-DA of all indexes are shown in Fig. S-F2a). It is seen clearly that the two first principal components are related to variation in θ_{D} and θ_{N}. Full cross-validation this purely linear "soft model" showed that the optimal number of principal components was eight. At this model rank, the cross-validated R^2 were: μ (0.02), θ_D (0.94), θ_N(0.64), p_D (0.47), $p_{N,}$(0.16), *PertSize* (<0.01) and *PertDir* (0.08). So obviously, parameters θ_{D} and θ_{N} were best predicted by the image analysis features of the results, based on this low-rank multi-linear PLS regression.

In order to simplify the interpretation of the correlation loadings, the score plot with typical examples is given in Fig. S-F2b, with some representative image examples superimposed. Here we see clearly that the different samples have clustered according to variation in the features. The diagonal going from bottom left to top right is associated with decreasing θ_{D} and increasing average value of N (from images with

no black to images with black dominating). This is supported by the object indexes describing when an image has just one object. In the lower left corner we for example see *CWDil* which implies that if white is the dominating cell type, giving very many objects in the original image, the expansion of these objects by adding pixels (i.e. dilation) is likely to generate one big connected region.

On the other hand, the diagonal going from upper left to bottom right is associated with decreasing θ_N and increasing local deviation from 3-periodic pattern. The latter is clearly seen in (b). The 3-periodic pattern is completely dominating the image in the top left. In the images on the opposite side of the diagonal, this pattern is still present, but is not nearly as dominant. In the top left we also see the index *C3Mean*. The clusters are sorted according to average area of all objects in the clusters, such that cluster 1 has the largest average.

Cluster 3 has relatively large objects coinciding with the fact that the large areas of 3-periodic pattern become connected regions under dilation. Studying the actual data shows that *C3Mean* attains maximum values (0.6 or over) for all the samples in the top left corner, and is less than 0.1 for all remaining samples.

Important parameters

The analysis so far has revealed that the most important parameters in determining the final state are the threshold values for Delta and Notch. That the two threshold parameters have a large influence on the dynamics is not a surprising result. However, we did expect that some of the other parameters would be more influential as well. In the different analyses, there is clearly some dependence on p_D, but the influence of the remaining parameters is almost negligible. There may be several explanations for this lack of influence. Obviously, one reason may be that the parameter simply does not have any major effect on how the system behaves. An alternative explanation is that the observed patterns may be highly influenced by factors not included in the experimental design, for example boundary conditions. Third, it may be that the chosen features do not capture the effect of the given parameter.

The parameters that seemingly do not have any influence on the patterning are μ and the parameters associated with the initial conditions. With respect to μ, this parameter is not present in any of the steady state equations, and thus, has in principle no influence on the pattern. It therefore seems natural that the features chosen here explain the variation in this parameter poorly.

Neither size of perturbation nor the direction of perturbation showed any strongly significant effects on the pattern formation, or *vice versa*. In relation to this, it should be mentioned that in the case of all cells being perturbed we simulated a total of 10 replicates for each of the parameter sets as each cell is randomly perturbed. In the analysis, these replicates behave similarly in the sense that they have approximately the same placement in the score plot (data not shown). Overall, these results indicate that there seem to be some robustness towards variations in initial conditions. Additional support comes from the fact that there are some similarities in pattern for the two types of perturbation. The dominant pattern is naturally quite different for the two, but with respect to the indexes describing greyscale, the models are more or less equal, and certain parameter combinations give a pattern very close to the 3-periodic pattern covering most of the lattice both when perturbing a single cell and all cells.

Choice of features

We cannot disregard that the lack of influence, poorly explained variance or robustness to initial conditions is due to lack of indices that would capture the effects of these factors. As mentioned above, this may be the case for μ, for which properties of the transient may have explained more of the variation in the parameter.

For the example model, we used features characterising the steady states. Most of these were fairly general, as we did not have much knowledge of the possible patterns before starting the analysis. The exception is the index describing the deviation from a 3-periodic pattern, as this pattern was predicted by a linear analysis. There are however, several other ways of generating general features describing an image than the ones presented here. A common feature for several of the results, as well as some of the indexes used here, is that they are not necessarily easy to interpret.

2.2.4 Conclusions from the explorative screening experiment

Fig. S-F2a) revealed systematic patterns of co-variation between the model parameters and the resulting image features. dominant latent variables (Fig. S-F2b) showed systematic clusters of sample images, related in an apparently systematic patterns. However, the statistical solution, based on combinations of standard image features and rather abstract cluster definitions, were not easy to interpret, be it perceptually or in mathematical, mechanistic terms.

However, the analysis indicated that the seven model parameters differed very much in how they could be predicted from linear combinations of the standard image characteristics. Rather than trying to enhance this modeling in terms of interaction effects etc, or expanding the experimental design to allow more dense sampling of the parameter space, we decided first to develop more a informative solution profiling method.

3. Sensory descriptive experiment

3.1 Spanning the parameter space by reduced factorial design

The third step is now to develop more dedicated and easily interpreted descriptors specifically for this system. An alternative approach is to take advantage of the human ability to detect and interpret patterns. The human visual system is a powerful system for parsing and assessing visual stimuli due to its highly parallel architecture and the brain’s apparent parallel processing. Thus, humans have a great capacity for analysing complex patterns, both in terms of the ability to identify different structures and shapes, and the ability to segment and interpret a pattern and report the results. The scientific approach of using human senses, including vision, to classify, describe, and analyse different objects, is termed *sensory science*.

Since sensory descriptive analysis involves human assessors, it will be more demanding and expensive than purely computational image analysis. So it is important to reduce the number of parameter combinations to be treated, with minimum loss of information. The previous screening research cycle indicated that the steady-state solutions were more strongly affected by some model parameters than by others. Hence, we decided to select a small, but informative subset of the previous

simulation conditions for sensory descriptive analysis, based on the technique of fractional factorial design. The previous screening design showed that particularly two of the above parameters (*PertSize*, *PertDir*) did not have much influence on the patterning of the final state. Based on this and an upper limit of 32 images for sensory analysis we designed a fractional 2^{7-2} factorial design. The main confounding pattern of the design is displayed in Table S-T4.

Within this fractional factorial design, there were 6 parameter combinations that did not destabilize the homogenous steady states. Having no pattern, these were removed from the set before analysis. Instead, we included two centre points times 3 replicates. Hence, a total of 32 images were submitted to the more demanding, but informative sensory descriptive analysis. Prior to the sensory analysis, the 50 x 50 lattice solution images were slightly blurred.

In addition, the same set of parameter combinations, integrated after having perturbed only at one single cell (instead of all cells), were also submitted to the sensory descriptive analysis. The conclusions from these were rather similar to those for images where all cells had been perturbed, so they will not be reported here.

3.2 Characterizing the solutions by human sensory profiling

A sensory descriptive analysis was carried out by the professional sensory panel at the Norwegian Food Research Institute, Matforsk, consisting of 11 trained assessors. The sensory laboratory used was designed according to guidelines in ISO standard, i.e. with individual booths, controlled ventilation system etc. First, the vocabulary for the descriptive analysis was developed in three steps, using both sample sets simultaneously. A total of 14 sensory terms were deemed necessary. Of these, two (*KernelSize* and *Circles*) pertained to the single-cell-perturbations only, and will largely be ignored here. The remaining 12 descriptors are defined in Table S-T5.

3.3 Exploring the results by multivariate soft modelling

The average of the panellists was taken as the 32 x 12 table of sensory results. This was submitted to various types of multivariate data modelling in order to search for possible systematic structures in the data. Standard multivariate soft modelling by bi-linear low-rank PLS Regression as used, for estimation, validation and display, as explained in Martens & Martens (2001) and implemented in The Unscrambler V9.6 (www.camo.com). The 7 model parameters and selected combinations of these were defined as regressands $\mathbf{Y} = [\mathbf{y}_1, \mathbf{y}_2, \ldots, \mathbf{y}_7, \ldots]$, and related simultaneously to the 12 sensory descriptors defined as regressors $\mathbf{X} = [\mathbf{x}_1, \mathbf{x}_2, \ldots, \mathbf{x}_{12}]$ via a few latent X-variables $\mathbf{T}_A = [\mathbf{t}_1, \mathbf{t}_2, \ldots, \mathbf{t}_A]$: $\mathbf{T} = \mathbf{X}\mathbf{V}_A$, where subscript *A* represents the number of latent variables $\mathbf{t}_1, \mathbf{t}_2, \ldots, \mathbf{t}_A$ deemed reliable by cross-validation (in this case $A = 3$, with the third one being small and pertaining to *MultiShade* only). The consecutive basis vectors of weight matrix $\mathbf{V}_A$ were defined according to the PLS criterion, as the eigenvector of the residual $\mathbf{X}'\mathbf{Y}$ that maximize the remaining X-Y covariance. The score vectors $\mathbf{T}_A$ were used for modelling both the model parameters and the sensory descriptors: $[\mathbf{X}, \mathbf{Y}] \approx \mathbf{T}_A [\mathbf{P}_A', \mathbf{Q}_A']$. This latent variables model may equivalently be reformulated in to a reduced-rank multivariate linear regression model, for simple prediction of $\mathbf{Y}$ from $\mathbf{X}$: $\mathbf{Y} = \mathbf{X}\mathbf{B}_A = \mathbf{X}\mathbf{V}_A\mathbf{Q}_A'$. Fig. 5 summarized the results of this PLS discriminant analysis. Simulation solution images lying close to each other in the PLS score plot were grouped together into classes, whose properties are summarized in Table S-T6.

Afterwards, the 32 x 12 table of sensory descriptors were submitted to a very different type of multivariate analysis, namely unsupervised hierarchical cluster analysis. Fig. S-F3 shows the result, which confirms that the 32 parameter combinations yielded solution images that from a sensory perspective fell into the distinct clusters or classes previously identified in Fig. 5.

Prediction of sensory descriptors

The analysis in the previous section gives an indication of the relations between sensory descriptors and computer-derived descriptors from the previous experiment. More information may be derived from a model in which the computer-derived descriptors are the predictors (**X**) and the sensory descriptors are the responses (**Y**). The significance of the regression coefficients **B** in the model $\mathbf{Y} \approx \mathbf{X}\mathbf{B}_A$ were estimated by jack-knifed PLSR, and studied graphically. The regression model predicts many of the sensory descriptors well, as judged by full cross-validation (M.Martens *et al.* in prep 2009).

As the sensory descriptors give a more interpretable classification of the samples than did the automatic mathematical filter-based image description, it would be desirable to perform a sensory analysis on the full set of samples. However, such an analysis is more time-consuming and expensive than evaluating the samples with computer-derived descriptors. An alternative approach is to develop a model for how to combine the computer- derived descriptors to the trained sensory panel within the set of 32 samples, and then use this to *predict* how the trained sensory panel would have evaluated the images not being part of the sensory analysis, from the computer-derived descriptors of these new images. Within the 32 of samples analyzed, this does seem feasible.

This prediction ability is illustrated in Fig. S-F4 for two new, “unknown” images not included in the sensory analysis set, along with their values of the sensory descriptors as predicted from the automatic mathematical filters. In these two images, sensory descriptor *Curls* is predicted to be high and low, respectively, and looking at the images this seems to make sense. It is also apparent from these images that *Curls* is positively associated with *Contrast*, *Sharpness* and *PatternBlack*. The two samples are clearly different, and the predicted sensory descriptors reflect these differences relatively well, suggesting that the computer-derived descriptors may be good predictors of the sensory descriptors.

A similar principle was also illustrated in Fig. 4: For a normal image whose parameter values are unknown, they can be correctly predicted from sensory profiling of the image. But the latter also shows that if the unknown image is of a new type not included in the calibration model, the parameter values predicted may be erroneous – but this unfortunate situation triggered an automatic outlier warning, so at least we get to know that there is something wrong.

3.4 Conclusions from the sensory descriptive experiment

The multivariate data modelling of the sensory descriptor space indicated that the various model parameter combinations generated solution images that could be grouped into distinct classes in the solution space. These could in turn be mapped back and forth into different regions of the parameter space by the “soft” multivariate PLS regression. Prediction of sensory descriptors in new, unknown images from their

automatic computerized image profiles also seems to work – as long as the images lie within the range of image qualities calibrated for.

With as few as 32 images, the spatial resolution of our sampling of the 7-dimensional parameter space is limited. We needed to check if the corresponding property space is smooth in between.

Moreover, some intriguing pattern types were discovered. For instance, while Fig. 5 showed that the sensory term *MultiShade* ("MS") was badly modelled by the first two PLS PCs, it was found to stand out with unique variance and hence dominated the third PC (not shown). When inspecting the images standing out in this third dimension, a recurrent pattern of "two-headed worms" was discovered (see e.g. Figs. 2D and 3C-D). Now we needed to study these and other details, to characterize them in more detail and to check under what conditions they arise.

4. Pursuit of discovered details

The fourth and final research cycle to be reported here was done with the intention to check the spatial smoothness and of the parameter/solution space mapping, and to pursue the newly discovered pattern types. In preparation for this research cycle, various aspects of boundary conditions and random initializations were explored.

Various cell lattice sizes were tested for various parameter combinations and initial perturbations. In general, lattice size had little effect on pattern appearance. But it was found that some large-scale patterns (e.g. Fig. 2B and S-F4b) characterized by high sensory scores of the descriptors *Straight Lines*, *Regular* and *Continuous* disappeared or were strongly reduced, when the cell lattice size was increased from 50 x 50 to 51 x 51. As mentioned in the main text, this indicates "crystal faults" between regions of purely 3-periodic patterns, positioned off-by-one; this is unavoidable in a system with non-integer (50/3) effects due to the continuous boundary conditions. Hence, the next simulation experiment was performed on 51x 51 lattices.

Different initial Delta-perturbations gave different positions of the spatial objects in the solution images, but with the visual appearance was generally unchanged. In order to allow more detailed quantitative comparison of solutions, the same set of 51 x 51 random initializations was used for all parameter combinations.

Only a subset of this experiment will be referred to here; the rest will be reported elsewhere.

4.1 Spanning the parameter space by dense sampling

The class III parameter combinations, $[\theta_D, \theta_N, p_D, p_N] = [0.7, 0.1, 10, 3]$ stood out with high values of the descriptor *PatternWhite* and *MultiShade* in the previous sensory experiment. Closer visual inspection of a few solution images revealed a local pattern of "worms with two heads" at (Fig. 3D, 4C) or around the former of these two conditions (Fig 4D), but not in the latter (Fig. 3C).

Hence the experimental design for the results reported here was set up to track the effect of moving through the parameter space the from the most similar class I-condition $[\theta_D, \theta_N, p_D, p_N] = [\mathbf{0.1}, 0.1, 10, 3]$, (Fig. 3A) to class III with two-

headedness (Fig. 3D) and beyond, by changing θ_D from 0.1 to 0.9 in small steps of 0.01; even denser sampling were included when deemed necessary due to rapid changes in the solution space. A subset of these many simulation solution images were submitted to sensory descriptive profiling.

4.2 Characterizing the solutions by human sensory profiling

The same sensory panel of 11 judges from The Norwegian Food Research Institute was engaged, a year after the previous experiment. In order to simplify the detection of spatial pattern details, the images were not smoothed this time. The sensory descriptor list (Table S-T5) was used almost unmodified; two new descriptor terms were needed to pick up new pattern types (Table S-T7).

4.3 Exploring the results by summary histograms and graphics

Fig. 7 showed how the solution image appearance changes in distinct jumps with increasing Delta threshold level. In that figure, the sensory panel average profiles were plotted for their first three their first principal components. For each sensory result, the corresponding θ_D level is plotted along a diagonal straight line; dotted lines connect the two type of data. For points with equally spaced values θ_D = [0.1,0,2,…,0.9] the sensory profile and the θ_D level are marked with red and blue squares, respectively.

For more detailed studies of the mechanisms involved, Fig. 7 compared the sensory profiles with statistical summaries of the protein levels. In Fig. 7b) the mean of the panel of 11 sensory assessors are shown for three of the sensory descriptors,. The vertical line segments illustrate the precision of the sensory results, connecting the two averages obtained when the panel was instead split into two sub-panels with 5 and 6 persons.

For each of the θ_D levels used in simulation, the relative frequency of Notch levels in the resulting 51 x 51 cell lattice was recorded. The diameter of the spots in Fig. 7a) represents the log of the number of cells with a given Notch level at parameter setting.

4.4 Conclusions

The peculiar image patterning (two-headedness, width of curls) discovered in the previous research cycle were now found to follow the non-linear dynamic model parameter θ_D a highly systematic, but even more peculiar pattern. At θ_D = 0.7, the center of the "*twoheadedness*"-region, four distinct Notch levels are observed. Moving θ_D up or down from 0.7 lead to bifurcations, increased computation time and loss of twoheadedness. *Thickness of curls*, on the other hand, reacted to other bifurcation regions.

6. Tables and figure

**Table S-T1: Overview of parameters in the system**			
	**Parameter**	**Description**	**Range (low,high)**
	Varied		
1	μ	Ratio between decay rates for D and N.	{0.5, 5}
2	θ_D	Threshold in Hill-function for D.	{0.1, 0.9}
3	θ_N	Threshold in Hill-function for N.	{0.1, 0.9}
4	p_D	Steepness in Hill-function for D.	{3, 10}
5	p_N	Steepness in Hill-function for N.	{3, 10}
6	M	Size of perturbation: percentage of value at homogenous steady state value.	{2, 20}
7	S	Direction of perturbation: less than or greater than homogenous steady state value.	{-1,1}
	Constant		
8	Variable of perturbation	Which variable to perturb from the homogenous steady state.	Delta ("D", as opposed to Notch, " N")
9	Lattice size	Number of cells in x- and y-direction.	50x50
10	Boundary condition	Definition of the set of neighbours for the cells at the boundaries.	Periodic

Table S-T2: The values of the threshold parameters (θ_D and θ_N) in the extended design. Cube 1 equals the original design.
All other factors in the design have values as described in Table 1.

	θ_D			θ_N		
	**Low**	**High**	**Center**	**Low**	**High**	**Center**
**Cube 1**	0.1	0.7	0.4	0.1	0.7	0.4
**Cube 2**	0.3	0.9	0.6	0.1	0.7	0.4
**Cube 3**	0.1	0.7	0.4	0.3	0.9	0.6
**Cube 4**	0.3	0.9	0.6	0.3	0.9	0.6

Table S-T3: Overview and description of extracted features.
See text for details.

Features	Definition	Interpretation
**Greyscale**		
DMean, DStd, DMax, DMin	Mean, standard deviation, maximum and minimum over all cells.	Final value of *D*.
NMean, NStd, NMax, NMin	Mean, standard deviation, maximum and minimum over all cells.	Final value of *N*.
DMinusNMean, DMinusNStd, DMinusNMax, DMinusNMin	Mean, standard deviation, maximum and minimum over all cells.	Difference between final levels of *D* and *N* in a given cell.
**Autocorrelation**		
ALMean, *ALp3corr*	Mean and closeness to 3-periodic pattern.	*L* denotes local: 4x4 matrix for shifts, $0 \leq m$, $n \leq 3$
AGMean, AGp3corr	Mean and closeness to 3-periodic pattern.	*G* den. global: 26x26 matr. for shifts , $0 \leq m$, $n \leq 25$
ADMean, ADp3corr	Mean and closeness to 3-periodic pattern.	*D* den. distant: 11x11matr. for shifts,$15 \leq m$, $n \leq 25$

**Object analysis**		
CiMean, *CiMax*	Mean and maximum over all objects in a given image.	Cluster number i = {1,…24};. Clusters are sorted according to area such that *C1* is the cluster with largest average area.
CWRaw, CWDil, CWErod<br><br>*CLRaw, CLDil, CLErod*<br><br>*CDRaw, CDDil, CDErod*<br><br>*CBRaw, CBDil, CBErod*	0 if the corresponding filter has more than one object; 1/16 (average over all possible filters) if the filter has one object.	Variable describing filters with one object. *Raw* is the original image, *Dil* is the dilated version and *Erod* is the eroded version. *W* corresponds to the white filter; *L* is light grey, *D* is dark grey and *B* is black.
Note: As described in the text there were 16 filters. However, the features *CWErodDil, CLErodDil, CDErodDil* and *CBErodDil* did not yield any new information when compared to the 12 other features. These features were therefore removed from the dataset in the final analyses.		

**Table S-T4: Main confounding pattern of the fractional factorial design**		
**Parameter**	**code**	**Confounding**
μ	A	A = EFG
θ_D	B	
θ_N	C	
p_D	D	
p_N	E	E = AFG
PertSize(S)	F	F = AEG
PertDir(M)	G	G = AEF

Table S-T5: Sensory descriptors			
**Name**	**Description**	**Low (1.0)**	**High (9.0)**
Whiteness	Average colour (NCS-system)	No white	White
MultiShade	How many shades of grey	No shades	Many shades
Contrast	How well the pattern is defined	Hardly	Clearly
Sharpness	Blurred, indistinct pattern	None	Clear
StraightLines	Presence of straight lines, direction is irrelevant	None	Many
PatternWhite	White pattern on dark background	No clear white pattern	Clear white pattern
PatternBlack	Dark pattern on light background	No clear dark pattern	Clear dark pattern
Curls	Presence of connected paths that cross	None	Many
Continuous	Degree of continuous regions	None	High
Regular	Degree of order	None	High
Associations	Degree of associations	None	Many
MentalLoad	Visual burden during analysis of image	None	High

Table S-T6: Characterisation of the four pattern classes. The + refers to a high value of the corresponding parameter; the – to a low value.

Class	Characteristics	Symbol	Defining parameter values	Example
I	Black patterns, curls	★	$[\theta_D -] =$ "*pI*"	Fig. 3a
II	Regular, straight lines	■ (yellow square)	$[\theta_D +, \theta_N +, p_D \neq p_N] =$ "*pII*"	Fig. 3b
III	White patterns, twoheadedness	● (blue circle)	$[\theta_D +, \theta_N -, p_D +] =$ "*pIII*"	Figs. 3d, 4c,d
IV	Highly complex, often multi-level patterns. With subclasses	◀ (cyan)<br>▶ (magenta)<br>▽<br>△	$[\theta_D +, \theta_N +, p_D +, p_N +]$<br>$[\theta_D +, \theta_N -, p_N +]$<br>Centre, initial value set # 1<br>Centre, initial value set #2	Fig. 3e<br>Fig. 3c<br>Fig. 3f

The + refers to a high value of the corresponding parameter; the – to a low value.

Table S-T7: Selected descriptors from the second sensory panel evaluation			
**Descriptor**	**Description**	**Low (1.0)**	**High (9.0)**
WidthCurls	Width of filaments	Narrow	Wide
Twoheadedness	Number of filaments with same features in both ends	None	Many

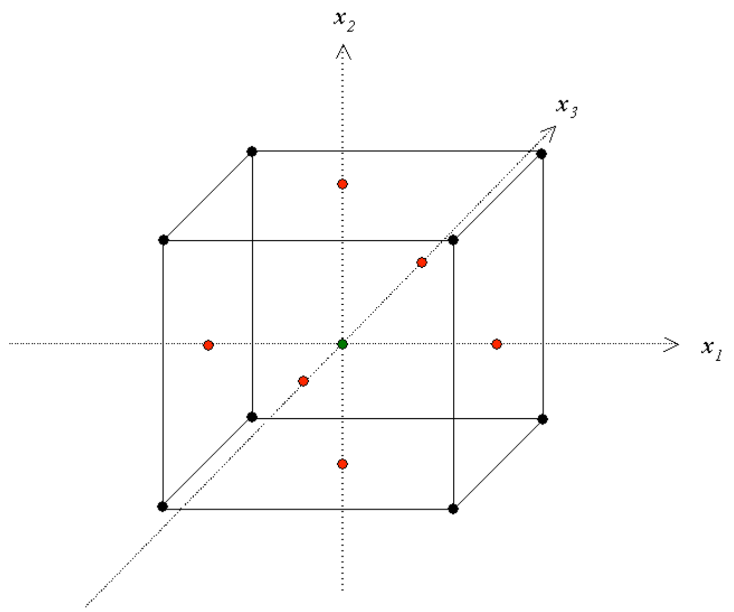

Figure S-F1: Illustration of a general 2^k factorial design, using k = 3. The factorial design consists of the eight corners (marked in black), in other words all combinations of high and low for the three variables x_1, x_2 and x_3. The factorial design is extended with a centre point (marked in green) and six axial points (at the faces of the cube, marked in red) in order to detect non-linear responses.

.

S-F2 a)

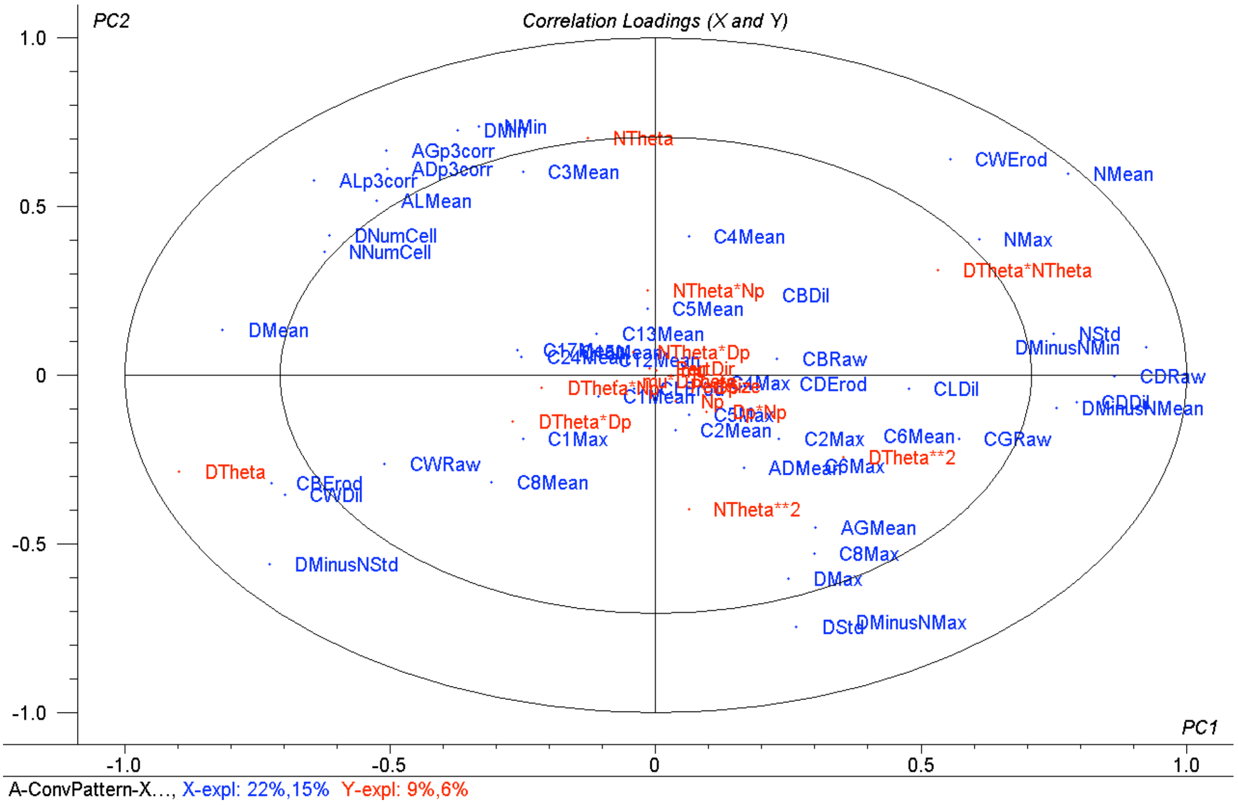
Correlation Loadings (X and Y)
PC2
PC1
1.0
0.5
0
-0.5
-1.0
-1.0
-0.5
0
0.5
1.0
NMin
NTheta
AGp3corr
ADp3corr
ALp3corr
C3Mean
ALMean
CWErod
NMean
DNumCell
NNumCell
C4Mean
NMax
DTheta*NTheta
NTheta*Np
C5Mean
CBDil
DMean
C13Mean
NStd
DMinusNMin
CBRaw
CDErod
CLDil
CDRaw
DTheta*Dp
C1Max
C2Mean
C2Max
C6Mean
CGRaw
CWRaw
DTheta
C8Mean
DTheta**2
NTheta**2
AGMean
C8Max
DMax
DMinusNStd
DMinusNMax
A-ConvPattern-X…, X-expl: 22%,15% Y-expl: 9%,6%

S-F2b)

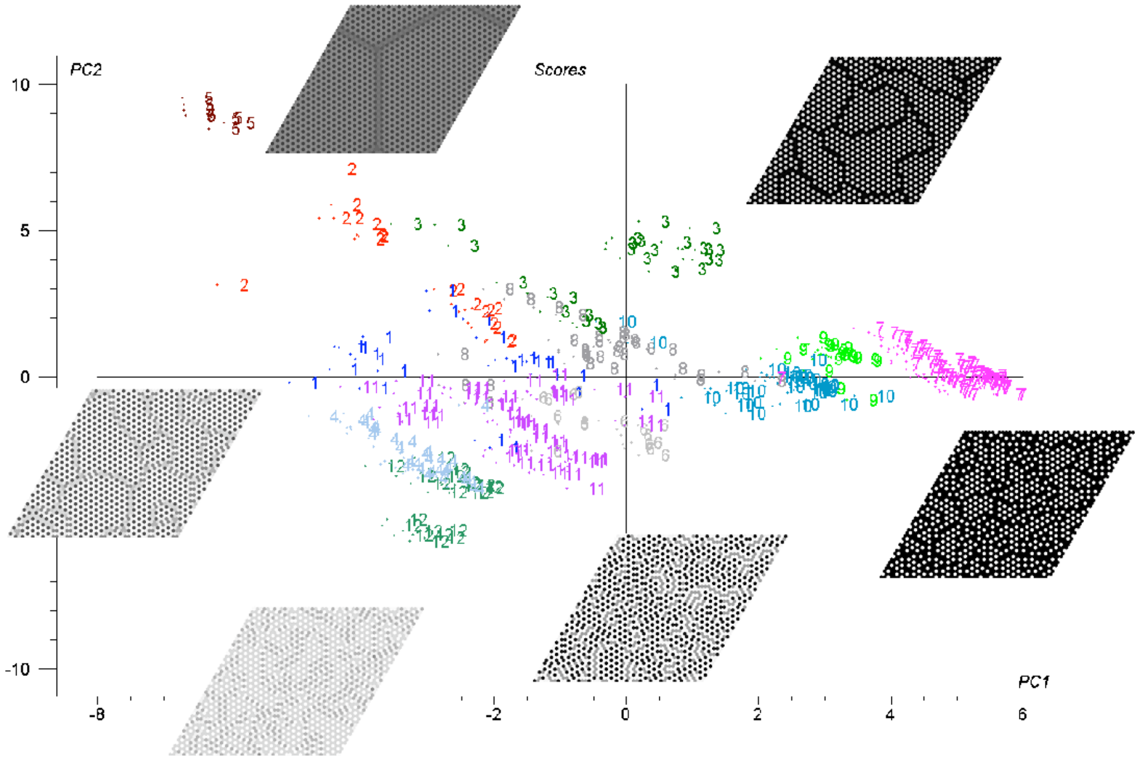

Figure S-F2: Combined PLS-DA analysis (i.e. all properties). (a) Correlation loadings. (b) Score plot (showing the samples along each model component) with typical examples. The samples are coloured according to the cluster they belong to. Both plots are showing the two first PLS components (the optimal number is 8). The threshold parameter θ_D dominates the first PC, while θ_N dominates the second PC. The features that explain the most of the variation are either related to greyscale or autocorrelation, although some of the features from the object analysis also contribute, mainly *C3Mean* (upper left), *C8Max* (lower right) as well as three of the extra clusters describing filters with only one object (*CWErod* (upper right), *CWDil* and *CBErod* (both lower left)). It is interesting to note that the indexes associated with autocorrelation, the most dominant being the local correlation with a 3-periodic pattern, lie approximately along the line of correlation with θ_N (upper left-lower right diagonal). This is seen clearly in (b) where the samples in the upper left cluster (cluster 5) all have a pattern that is dominated by this pattern. Here $\theta_N = 0.7$. In the samples in the lower right corner this pattern is still present, but is not nearly as dominant. Here $\theta_N = 0.1$. Along the other diagonal (along the line of correlation with θ_D) it is greyscale that is the dominant feature – going from low mean value of the final values in the lower left corner to high mean value in the upper right corner, associated with a greater dominance of black cells (i.e. cells with a high level of *N*).

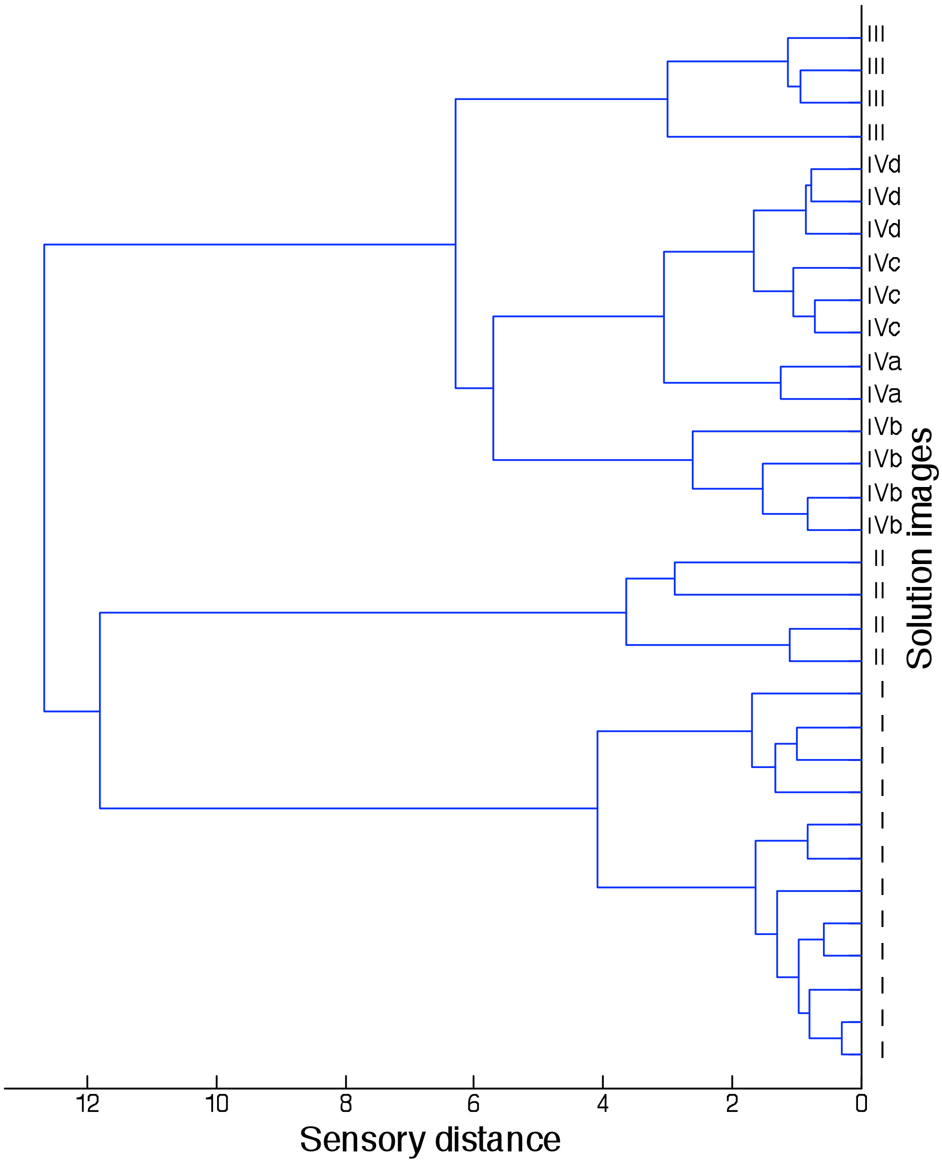

Figure S-F3 Grouping of the sensory panel averages: Hierarchical cluster analysis of the 32 solution images of the selected parameter combinations, based on Euclidian distances in over the 12 sensory descriptors. Branches here named c1 and c2 correspond to classes I and II in Figure 5a).

On the basis of this analysis, the 32 parameter combinations were ascribed to various classes. IVc and IVd represent two centre points with opposite perturbation directions, analysed in three sensory replicates.

(a)

(b)

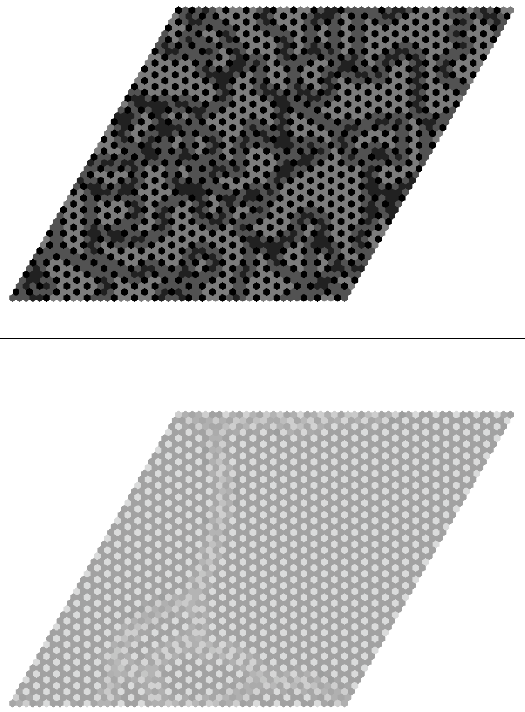

(c)

	(a)	(b)
Whiteness	2.54	5.16
MultiShade	4.41	4.15
Contrast	7.98	3.54
Sharpness	7.72	3.54
StraightLines	2.66	4.48
PatternWhite	1.44	2.36
PatternBlack	7.53	3.63
Curls	7.69	0.38
KernelSize	1.06	1.34
Circles	1.35	1.13
Continuous	3.78	5.96
Regular	1.89	4.92

Figure S-F4: Example of predicted sensory descriptors from automatically computed mathematical filters, illustrated for two new, "unknown", slightly blurred solution images (a),(b). The predicted values of the sensory descriptors are given in the left and right column in (c) respectively, with the highest value for each descriptor highlighted.
